# Cities for global health

**DOI:** 10.1136/bmj.k3794

**Published:** 2018-10-03

**Authors:** Majid Ezzati, Christopher J Webster, Yvonne G Doyle, Sabina Rashid, George Owusu, Gabriel M Leung

**Affiliations:** 1 School of Public Health, MRC-PHE Centre for Environment and Health, WHO Collaborating Centre on Non-Communicable Disease Surveillance and Epidemiology, Imperial College London,; 2 Faculty of Architecture, The University of Hong Kong, Hong Kong, China; 3 Public Health England, London, UK; 4 James P Grant School of Public Health, BRAC University, Dhaka, Bangladesh; 5 Centre for Urban Management Studies, Institute of Statistical, Social and Economic Research (ISSER), University of Ghana, Accra, Ghana; 6 LKS Faculty of Medicine, The University of Hong Kong, Hong Kong, China

## Abstract

In the first of a new series of articles on the role of cities in health, **Majid Ezzati and colleagues** call for greater action to reduce health inequalities within cities

The number of people, and proportion of the world population, living in cities has increased steadily, with 4.2 billion urban residents now accounting for 55% of the world’s population (fig 1). That urban living influences health is well recognised and increasingly included in broader discussions about cities and sustainable human development. The general tone of such discourse, however, tends towards the negative aspects of infectious outbreaks, vehicular pollution, waste disposal, and unhealthy lifestyles^2^ rather than the “positive and progressive aspects of cities . . . recognised by historians, economists, and other social scientists.”^3^

**Fig 1:**
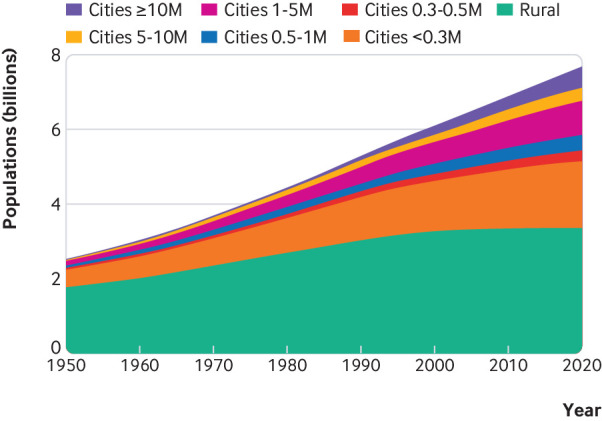
Number of people living in rural areas and in cities in the world. Data are from the World Urbanization Prospects^1^

Empirical evidence strongly points to urban residents having better health than their rural counterparts since at least the early to mid 20th century, in high income as well as low and middle income countries.^3^ ^4^ ^5^ ^6^ The health advantages of urban living, however, are unevenly distributed in cities, with massive inequalities existing over short distances (fig 2).^4^ ^7^ ^8^ ^9^ ^10^ ^11^ Our urbanising world provides an opportunity, and an imperative, to not only further improve population health in cities but also to leverage cities as nodes in a natiotrafinal and global network to improve health in and across countries. Reducing inequalities is fundamental because population health suffers where inequalities are larger.^12^ ^13^

**Fig 2:**
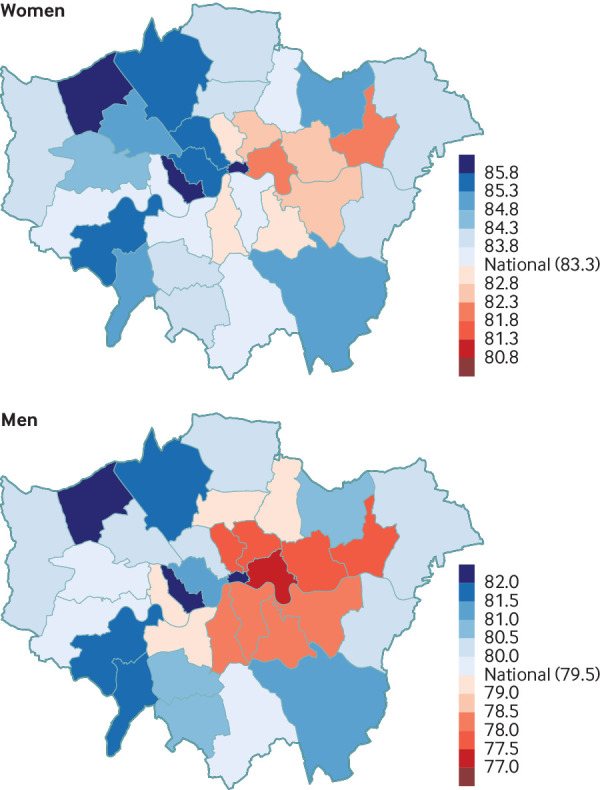
Life expectancy at birth in 2012 in London’s local authorities. Data are from Bennett et al^7^

The urban health literature commonly uses the “healthy city” concept to frame discussions.^14^ ^15^ The idealised healthy city, although aspirational, can easily be disconnected from the complex dynamics of urban development, in which cities’ demographics and social, natural, built, and food environments are constantly changing through interactions between individual, corporate, and public actions. Limited attention has also been given to the essential role of urban services, including healthcare, childcare, and public safety. Thus the policy challenge for improving health in cities, first laid out a century ago by Chapin,^16^ remains—to identify and implement institutional and technical innovations in every sector that form transition pathways to better health, taking into account the contemporary local social, demographic, and economic conditions. We discuss a set of themes in which municipal governments and administrators (referred to as “cities” hereafter) can foster innovation in technology and practice and achieve economies of scale in services that improve the health of their own residents and benefit a wider geography, with emphasis on their role in reducing health inequalities.

## Defining the role of cities in global health

### Environment

The infrastructures, technologies, and regulations that collectively provide clean sanitation and water have been a cornerstone of health improvement in cities for centuries.^16^ ^17^ ^18^ Although much recent attention has been on water quality, many cities face the additional challenge of severe water shortages due to inefficient management and unfavourable natural or human induced hydrological cycles. Shortages have led to water rationing and rising water costs, which disproportionately affect poor people. Water resource management (especially allocation among agricultural, industrial, and human use) often goes beyond the jurisdiction of individual cities, but cities can incentivise and encourage the use of technologies for more efficient and robust use of water resources. These include storm water collection and drainage, distributed or on-site treatment of wastewater, and source separation of human waste.^19^

Other urban environmental factors that affect health include air and noise pollution, green space, and the overwhelming volume of general solid waste, as well as electronic, battery, industrial, and other toxic waste. Cities can reduce pollution through infrastructure planning and regulations that change energy or transport technologies and behaviours. But the inequality challenge remains, as poorer areas are often designated to accommodate waste from richer areas of the same city or even from other locations, some in different continents.^20^

### Housing

**Fig 3:**
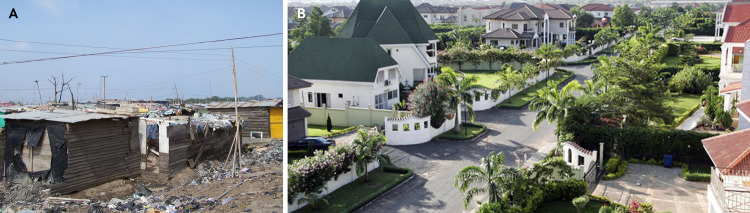
Homes in poor (A) and wealthy (B) neighbourhoods of Accra, Ghana, showing the extent of inequality in housing and the living environment just kilometres apart

Housing affects health through both social (interaction versus isolation) and physical (temperature, moisture, mould, pollutants, sunlight, and crowding) environments.^21^ The agglomeration benefits that attract people to cities inevitably create higher living densities and housing costs, which in larger cities are exacerbated by the presence of highly paid expatriate staff employed by multinational corporations. The high cost of housing in cities leads to inequalities in housing quality and neighbourhood conditions (fig 3).^22^ It also reduces the income that people have available for food, healthcare, energy, education, and leisure or limits the time that people can spend on these because they commute longer distances. In rapidly growing cities, slums have emerged as homes for millions of urban poor people who are priced out of the formal housing market and live in crowded, windowless, and flimsy structures without adequate sanitation and other essential services.^23^ ^24^

Cities have traditionally cleared slums and redeveloped these neighbourhoods. Unless accompanied by housing policies that support poor people, however, such actions can push poor families to farther locations and can affect their job opportunities and access to services such as education and healthcare.^25^ Slum upgrading, through equitable land tenure, construction of sanitation, water, electricity, and road infrastructure, and provision of essential services, has the potential to improve the health of slum residents without displacing them, but must overcome the political influence of powerful elites who benefit from rents on slum dwellings and from the sale of land for private development.^25^ ^26^ ^27^ ^28^ More broadly, city governments can tackle housing challenges through high quality state owned social housing and through fiscal policies and regulation that incentivise housing associations, public-private partnerships, or private entities to develop safe and healthy housing that operates at low cost and in the interest of low income people.^29^ ^30^ ^31^

### Nutrition

**Fig 4:**
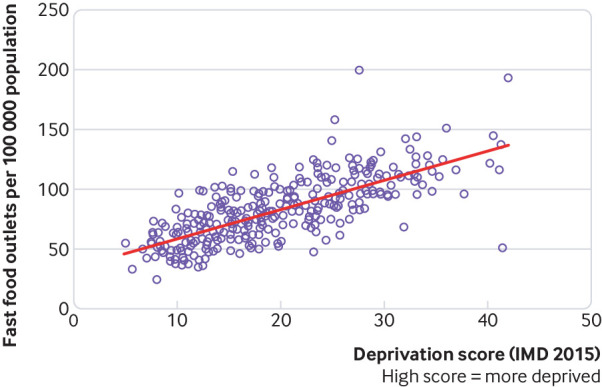
The association between deprivation and density of fast food outlets across England’s local authorities (source: Public Health England)^32^

Although urban living is often taken as a proxy for unhealthy eating, cities provide opportunities for better nutrition.^5^ Infrastructures such as roads and electricity facilitate the trade, transport, and storage of food, which can reduce the effect of agricultural shocks and seasonality and enhance dietary diversity. At the same time, the commercial nature of food provision in cities can raise the cost of healthy foods and enable transnational and local food industries to market unhealthy foods. This is especially true in poor and marginalised communities (fig 4), where a combination of cost and limited time and space for cooking healthy meals leads to poor nutrition.

Cities can leverage the benefits of food trade and sales through food hygiene laws and inspections and through healthy food programmes that support poor people, such as healthy school meals, food stamps designated for healthy foods,^33^ requiring the availability of healthy foods such as fruits in grocery stores, and restricting the marketing and sales of unhealthy foods. Cities can also use their planning and zoning powers to facilitate access to land and water for urban agriculture, which can improve food security and nutrition.^34^

### Addiction

Cities provide a focal point for the distribution and consumption of addictive substances (tobacco, alcohol, and illicit drugs).^35^ Both commercial and informal suppliers typically target poor and vulnerable communities.^36^ Cities have an important role in tackling addiction and its health consequences^37^; they can, for example, levy additional taxes beyond national or provincial dues; restrict the locations and opening times of alcohol and tobacco outlets through licensing; regulate product advertising; legislate smoke-free areas; raise the minimum legal age for sales; institute sobriety checkpoints and random breath testing; implement designated driver campaigns; sanction the use of currently illicit substances in monitored locations for harm reduction; and provide addiction counselling and treatment services.^38^

### Universal health coverage

Universal health coverage has emerged as a priority for national and international health agencies. Although financial protection and limiting out-of-pocket payments often comes under national or provincial jurisdiction, some cities provide additional safety nets for those without insurance coverage.^39^ More importantly, health services in cities both provide care to their own residents and act as referral hubs for rural residents who need specialist care because the higher population density and reduced distances in cities facilitate regular and frequent interaction with primary health services, and provide economies of scale for secondary and tertiary care.^39^ Achieving universal coverage and enhancing health equity requires careful planning of service location and operating times to serve low income families with long and inflexible work hours and to reverse the current pattern of more affluent groups getting the benefits of high quality urban health services.^40^ ^41^ ^42^ ^43^ Finally, city living might isolate vulnerable groups, including elderly people, with no local social networks and limit or delay their use of health services. Compensating mechanisms include an integrated local primary health and social care system and combining new sensing and communication technologies with key health workers to ensure interactions are made in good time to prevent severe declines in health.

### Public safety and emergency response

Public safety and emergency response are essential functions of cities that, under normal conditions, can contribute to health through prevention (enhanced neighbourhood and traffic safety and reduction in crime) and through mitigating adverse outcomes from acute events (faster response to heart attacks, road traffic crashes, and fires). Achieving these objectives requires sustained investment in infrastructure and personnel, including street lighting, fire services, ambulances, police, and paramedics, as well as training and oversight to ensure equal treatment of citizens. Extreme events can overwhelm local services and thus require an agreed contingency plan that includes a well rehearsed command structure, a communications strategy, recovery management, and a good understanding of the role of governmental agencies and civil society, which should all be established before the crisis arises.

### Infectious disease outbreaks

Because of their population concentration and connectivity, cities are a rate enhancing or rate limiting gateway to infectious disease transmission, with consequences that extend beyond health, as seen recently with Ebola. This makes detection and control of epidemics a direct function of cities, which requires robust city based outbreak surveillance, detection, and control systems that are coordinated between neighbouring municipalities. Non-drug interventions are often the first—and sometimes the only (especially in resource poor settings)—line of defence against outbreaks. Quarantine, isolation, and contact tracing are almost always carried out by municipal public health authorities. Cities also have a major role in the distribution of antiviral drugs, for prophylaxis and for treatment, and vaccination campaigns, both of which featured prominently in the 2009 H1N1 pandemic.^44^ Finally, an important route for introduction of infections in cities is when infectious agents cross the species barrier in wholesale and retail markets, initiating a cascade of events that ultimately seed an epidemic.^45^ Limiting such events requires a “one health” approach that recognises the critical interface between animal and human health and extends to agriculture, aquaculture, and animal husbandry practices.

### Smart cities and emerging economies and technologies

Advances in sensing, computing, and communication technologies are creating unprecedented opportunities, as well as challenges, to improving urban health and reducing inequalities. Examples include the use of digital footprints for tracking disease and mobile phones for health information and alerts; distributed sensor technologies to detect water and air pollution, mould, traffic flows and crashes, and crime; better monitoring of, and response to, health of newborns and elderly people through personalised sensing; better nutrition through online shopping and home delivery; and more active or more efficient transportation through bicycle and car sharing systems and eventually autonomous vehicles. Such technologies also have the potential to worsen health and widen inequalities. Sharing systems like Airbnb may be affecting the already limited housing supply in cities, and the gig economy may be worsening social inequalities by reducing wages and job security.^43^ ^46^ Home delivery of goods and services and diversion of traffic to reduce congestion could increase air pollution and the risk of traffic related injuries in residential areas, and reliance on online shopping may increase social isolation. Individual cities cannot stop such trends but will need to carefully monitor their penetration and impact and be prepared to intervene through agile legislative, regulatory, and fiscal policies to maximise benefits and minimise harms, especially in terms of inequalities.

### Migrant, transient, and peri-urban populations

Cities around the world are homes to tens of millions of refugees, asylum seekers, undocumented migrants, and internally displaced persons. City boundaries and residents are also increasingly blurred by large groups of transient populations who seek jobs in cities, even in tightly controlled systems such as the Chinese “hukou” (household registration) system, and by large peri-urban communities. These groups and areas are functionally part of the city but are often administratively hidden and not entitled to full land and residency rights or to services such as waste collection, home water connections, social insurance, and healthcare, which worsens social and health inequalities in cities.^47^ Agricultural based industries and off-farm activities that generate sustainable income and better rural infrastructure and services can slow the rural-urban migration.^48^ But overcoming these inequalities in cities can be achieved only by city administrations acknowledging the presence, contributions, and needs of migrant, transient, and peri-urban populations and by providing equitable access to quality healthcare and promotion of rights to safe accommodation and working environment.

## Conclusions

The concentration of knowledge and innovation, economic activity, healthcare, education, and other public services endows cities with the potential to deliver substantial improvements to the health and wellbeing of their residents and those of other parts of the country.^16^ ^37^ Further, the local politics in cities, whereby politicians and citizens live side by side as members of the same community, provide an opportunity to avoid and resist the exclusionary and austerity trends seen in national politics and economics around the world and to make health inequalities the central focus of urban health policies. A challenge to this, described a century ago by Chapin and equally relevant today,^16^ is the fragmented administrative and technocratic systems in cities. When coupled with pressure from various interest groups, these can easily lead to either continuing cities’ own past choices or replicating those elsewhere. To overcome this inertia and harness the health enhancing potential of cities requires using the cross sectoral roles of mayors and city councils to build health and health equity in all policies. Beyond individual cities, global and regional city networks (such as United Cities and Local Governments https://www.uclg.org/ and the C40 network https://www.c40.org/) provide an opportunity for shared learning and coordinated experimentation of innovative policies and how these can be adapted to contemporary local social, demographic, and economic conditions. Building on this thinking, *The BMJ* is launching a series of articles on important themes in urban health, such as emerging economies and technologies; extreme events and emergencies; housing; migration; and water resource management. The series will focus on actions that cities can take to reduce health inequalities and deliver on their potential to create better and healthier lives for all.

## Notes

Contributions: ME and GML developed the manuscript concept and wrote the initial draft. All other authors contributed to contents and writing. ME is the guarantor.

Provenance and peer review: Commissioned; externally peer reviewed.
